# The Impact of PPAR**γ** Genetic Variants on IBD Susceptibility and IBD Disease Course

**DOI:** 10.1155/2012/349469

**Published:** 2012-02-14

**Authors:** Jessica Mwinyi, Christa Grete-Wenger, Jyrki J. Eloranta, Gerd A. Kullak-Ublick

**Affiliations:** ^1^Department of Clinical Pharmacology and Toxicology, University Hospital Zurich, Raemistrasse 100, 8091 Zurich, Switzerland; ^2^Division of Gastroenterology and Hepatology, University Hospital Zurich, Raemistrasse 100, 8091 Zurich, Switzerland

## Abstract

PPAR*γ* is a nuclear receptor that regulates numerous pathways including cytokine expression and immune responses and plays an important role in controlling colon inflammation. We aimed at determining the occurring *PPARγ* SNPs, at predicting the haplotypes, and at determining the frequency outcome in inflammatory bowel disease (IBD) patients in comparison with healthy controls. We determined genetic variants in the coding exons and flanking intronic sequences of the *NR1C3* gene in 284 IBD patients and 194 controls and predicted *NR1C3* haplotypes via bioinformatic analysis. We investigated whether certain *NR1C3* variants are associated with susceptibility to IBD or its disease course. None of the detected 22 *NR1C3* variants were associated with IBD. Two variants with allelic frequencies over 1% were included in haplotype/diplotype analyses. None of the *NR3C1* haplotypes showed association with IBD development or disease course. We conclude that *NR1C3* haplotypes are not related to IBD susceptibility or IBD disease activity.

## 1. Introduction

Crohn's disease (CD) and ulcerative colitis (UC) are chronic recurrent forms of inflammation of the gastrointestinal tract (inflammatory bowel disease, or IBD), which are characterized by an onset in young adulthood and by an unpredictable disease course that may lead to debilitating complications [1]. The combined prevalence of CD and UC is estimated to be 100 to 200 per 100 000 individuals in developed countries [2]. IBD is thought to be of multifactorial genesis including a complex interplay among genetic, environmental, microbial, and immune factors [3]. The exact molecular pathogenesis of IBD is not yet fully elucidated [4]. Although great advances have been made in the clinical management of IBD, curative therapeutic strategies do not exist.

It has been recognized that components of pro- and anti-inflammatory signalling cascades seem to play an important role in the pathogenesis of IBD. Initially, pro- and anti-inflammatory cytoplasmic receptors that are activated by bacterial lipopolysaccharides, such as nucleotide oligomerisation domain (NOD) 2/caspase recruitment domain (CARD) 15, and NOD1/CARD4, have been especially studied in the past and have been identified as IBD susceptibility genes. These findings emphasized the pivotal role of an interaction between enteric microbes and the intestinal immune system in the pathophysiology of IBD [5–8].

Current evidence suggests that an additional receptor, PPAR*γ*, plays an important role in the regulation of colon inflammation. PPAR*γ* belongs to the nuclear receptor family that consists of approximately 50 different transcription factors, which are known to be involved in the regulation of a wide range of different biological processes. PPAR*γ* controls the expression of a large number of different genes and was initially identified as an important regulator of genes involved in lipid metabolism and insulin sensitisation [9]. PPAR*γ* acts through heterodimerisation with another nuclear receptor, retinoid X receptor *α* (RXR*α*). The heterodimer binds to specific DNA response elements within the promoters of its target genes (peroxisome proliferator response elements, PPREs) [10]. PPAR*γ* is mostly expressed in adipose tissue and the large intestine. Kidney, liver, and small intestine express intermediate levels, whereas PPAR*γ* is barely found in muscle [11].

PPAR*γ* is known to modulate the expression of key transcription factors and kinases involved in inflammatory signalling cascades such as NF-*κ*B, c-Jun, c-Fos, and nuclear factor of activated T cell (NFAT). Thereby, PPAR*γ* is able to inhibit the mucosal production of proinflammatory cytokines, such as interleukin 1*β* (IL-1*β*) and tumor necrosis factor-*α* (TNF*α*), and to downregulate the expression of various adhesion molecules [12, 13]. Based on these findings, it has been demonstrated in mouse models that activation of PPAR*γ* leads to an efficient reduction of the severity of intestinal inflammation by suppressing excessive immunoinflammatory responses [14, 15]. As a consequence, PPAR*γ* is currently under investigation as a potential target for novel anti-inflammatory agents [9]. Because of its central role in the regulation of colon inflammation, we hypothesized that PPAR*γ* could be a putative susceptibility gene for the development of IBD. The *PPAR*γ** gene *NR1C3* is located on chromosome 3 and is composed of 9 exons. Alternative splicing yields three different protein isoforms, PPAR*γ*1, PPAR*γ*2, and PPAR*γ*3, which differ in the amino acid composition at their 5^′  ^ends. The isoform PPAR*γ*2 is the most abundant PPAR*γ* protein found in a number of human tissues [11]. PPAR*γ* is known to be polymorphically expressed. Several SNPs have been described, one of which has been shown to have consequences for both the conformational protein structure and protein function [16, 17]. So far, only a few studies have assessed the role of a few discrete *NR1C3* gene polymorphisms in IBD pathogenesis. A systematic study to comprehensively investigate the role of global polymorphic features of the *NR1C3* gene with a focus on its role in IBD susceptibility, such as the one presented here, has not been previously performed.

In the present study, we aimed at determining all occurring mutations and SNPs in the exonic regions of the *PPAR*γ** gene *NR1C3*, at bioinformatically predicting the arising haplotypes, and at evaluating their association with the risk to develop IBD and with IBD activity in a well-sized cohort of IBD patients and non-IBD controls. 

## 2. Materials and Methods

### 2.1. Patients

Two hundred and eighty-four clinically diagnosed Swiss IBD patients (140 UC and 144 CD patients) were recruited at the centers participating in the Swiss Inflammatory Bowel Disease Cohort Study (SIBDCS) [18]. All patients gave their informed consent to the inclusion into the study. An ethical approval was obtained from the Medical Ethical Committees of the University Hospital Lausanne, Switzerland, and all local study sites. EDTA blood samples were stored at the central tissue repository at the Institute of Pathology, University of Bern, Switzerland. The SIBDCS data center at the University Hospital of Lausanne, Switzerland, provided data on demographics and past and current disease characteristics. Diagnosis of IBD (CD or UC) was confirmed by the study investigations based on clinical presentation, endoscopic findings, and histology. Demographic and clinical information on IBD patients is summarized in Table 1.

### 2.2. Control Subjects

One hundred and ninety-four non-IBD controls were recruited from gastroenterological patients undergoing surveillance colonoscopy, who did not show any symptoms of IBD. History of colorectal cancer was used as an exclusion criterion for both IBD patients and non-IBD controls. All subjects provided their written informed consent to be included in the study. Ethical approvals were obtained from the local medical ethical committees of all study sites involved in the collection of non-IBD samples.

### 2.3. Sequencing Reactions

DNA was extracted from EDTA-blood or intestinal biopsies using the QIAcube robotic workstation and a standard procedure (QIAamp DNA Mini Kit, QIAGEN, Switzerland). The PCR and sequencing primer design was based on the NCBI reference sequence for *NR1C3* (GenBank accession number NT_022175.18). Primers for genomic DNA were designed to span all 7 exons expressed in case of the most often occurring PPAR*γ* protein variant 2 including at least 50 bp of flanking intronic sequences at both 5′ and 3^′  ^ends. The DNA sequences of purified PCR fragments were obtained with an ABI 3730xl sequencing machine. Details of the PCR primers can be found in the Supplemental Table 1 available online at doi:10.1155/2012/349469. Optimized PCR conditions, and methods used for subsequent purification, and sequencing of the fragments are available upon request.

### 2.4. Statistical Analysis of Allele Frequencies and Genotype Distributions

To detect differences in SNP distribution between case and control groups or between disease activity groups, the Chi-Square test or the Fisher's exact test was used. A *P*  value of <0.05 was considered as significant in noncorrected statistical tests and of <0.0023 after correction for multiple testing of 22 variants (according to Bonferroni). Differences in CDAI (Crohn's disease activity index), mtwsi (modified Truelove Witts severity index), or CRP (C-reactive protein) means in dependency of the genotype were calculated using the Mann-Whitney *U* test. The statistical analysis was performed using the software package SPSS 19 (SPSS Inc., Chicago, IL).

### 2.5. Haplotype and Diplotype Analysis

The FAMHAP software was used to calculate the haplotypes and diplotypes based on the detected SNPs and mutations in the *NR1C3* gene and to detect differences in haplotypes and diplotype distributions in case and control groups. FAMHAP performs a permutation test on associations between estimated haplotypes and the affection state based on Monte Carlo simulations. A value of *P* < 0.05 was considered to be significant. Haplotype and diplotype calculations were performed on 256 IBD patients and 148 non-IBD controls, from which all sequence data of adequate quality were obtained. To allow referral to specific haplotypes, a frequency-based priority criterion was used to name haplo- and diplotypes (e.g., *H_1 or D_1* for the most often occurring haplotype or diplotype, Table 8).

### 2.6. Calculation of Linkage Disequilibria

 Linkage disequilibria (LD) were calculated using the *r* ^2^ statistics. Calculations were performed using the software package Haploview (http://www.broadinstitute.org/scientific-community/science/programs/medical-and-population-genetics/haploview/haploview).

## 3. Results

### 3.1. NR1C3 Sequence Variability

DNA samples from 284 IBD patients (CD or UC) and 194 healthy controls were initially sequenced for the seven *NR1C3* splice variant 2 coding exons including at least 50 bp of the neighbouring intronic sequences. The sequencing results of 256 IBD patients (126 UC and 130 CD patients) and of 148 healthy controls were of adequate quality and complete for all seven exons to be further used in haplotype analyses. Table 1 shows the demographic data of the individuals included into the* NR1C3* analysis.

The sequence data were screened for genetic variation in the *NR1C3* gene, using the Basic Local Alignment Search Tool (BLAST; http://www.ncbi.nih.gov) and the GenBank entry NT_02257.18 as the reference sequence. As shown in Tables 2 and 3, altogether 22 variants were detected, which—with exception of one mutation (one individual was found to be homozygous for variant number 6)—were in Hardy-Weinberg equilibrium. The majority of variants were single-nucleotide substitutions. Only one variant was characterised by a base-pair insertion leading to a frame shift. The majority of variants have not yet been described in the NCBI SNP database. Nine variants were found in exonic regions, 11 variants were found in intronic regions, one variant was detected in the 5′-prime region, and 1 variant was found within the 3′ end of *NR1C3*. Only two of the detected variants (no. 1 and no. 7, Table 2) lead to nonsynonymous amino acid exchanges within the PPAR*γ* protein. Furthermore, only two variants occurred with an allelic frequency of more than one percent (*rs1801282 *and *rs3856806*), thus fulfilling a definition of a genetic polymorphism (Table 3).

**Figure 1:**
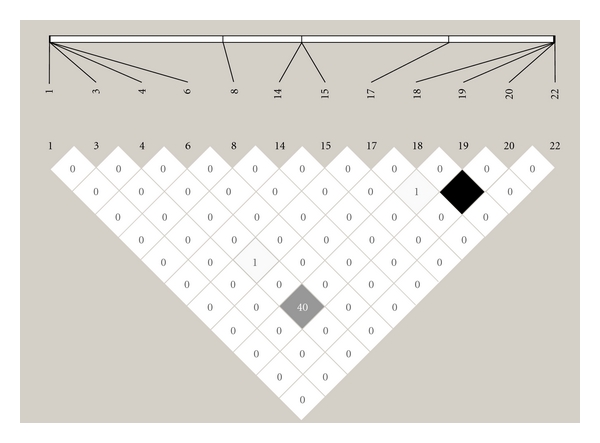
The LD plot for all *NR1C3* variants that were found in the non-IBD control group. *r* ^2^ = 0 (white), 0 < *r* ^2^ < 100 (shadows of grey), and *r* ^2^ = 100 (black). Numbers within squares: *r* ^2^ values (0–100%), LD: linkage disequilibrium.

The two most often occurring variants *rs1801282 *and* rs3856806* were found to be in moderately strong linkage (Figure 1, *r* ^2^ = 40%, SNP numbers 4 and 19) This finding is in good agreement with previous publications [19–21]. With the exception of two individuals, who were carriers of mutation numbers 18 and 20 or 7 and 10 in combination, no subject ever carried more than one rare *NR1C3* variant.

### 3.2. Distribution of Allele and Genotype Frequencies within IBD Patient and Healthy Control Group

As shown in Table 3, only two variants (*rs1801282 *and *rs3856806*) occurred in an allele frequency higher than 1% in IBD and non-IBD control group. No significant differences in allele frequencies were observed.

Table 4 shows the results when comparing the frequency of *NR1C3* genotypes carrying distinct genetic variants in heterozygous or homozygous form in IBD patients and non-IBD controls. No significant differences in the distribution of variant carriers were observed. In case of the often occurring SNPs *rs1801282 *and *rs3856806,* we investigated also the variant carrier frequency outcome after stratification according to disease subgroup (CD or UC) and different disease activity parameters, such as fistula state, occurrence of extraintestinal manifestations (EIMs) and overall clinical disease activity as evaluated by the disease activity indices CDAI and mtwsi. As demonstrated in Tables 5, 6 and 7, none of the mentioned factors were significantly associated with the occurrence of variants *rs1801282 *or* rs3856806*.

Furthermore a comparison of the mean values of leukocyte or CRP concentrations in plasma did not show any significant differences between IBD patients carrying the variant *rs1801282 *or* rs3856806 *and non-variant carriers within the patient group (data not shown).

### 3.3. Haplotype and Diplotype Analysis

The two *NR1C3 *genetic variants *rs1801282 *and* rs3856806, *which occurred in an allele frequency of higher than 1%, were included in the bioinformatic haplotype prediction using the computer programme FAMHAP. For this analysis, all individuals were considered, for which the sequencing outcome of all 22 variant loci was complete. Thus, it was possible to include 256 IBD patients (126 UC and 130 CD patients) and 148 controls. As shown in Table 8, four haplotypes (H1 to H4) were predicted to be in best reconstruction for both cohorts leading to eight different putatively occurring diplotypes (D1 to D8). All haplotypes and five diplotypes were predicted to occur at a frequency higher than 1% in the non-IBD control group.

Neither the overall haplotype nor the overall diplotype pattern varied significantly between the IBD group (or the IBD subgroups) and the control group. The result remained non-significant when investigating a possible relationship between the occurring haplotype or diplotype distribution pattern and disease activity (abundance of EIMs, fistulas, or high activity indices; Tables 9–11).

## 4. Discussion

PPAR*γ* is an important modulator of pro- and anti-inflammatory signalling cascades involving NF-*κ*B. The importance of PPAR*γ* is underlined by the fact that efficient anti-inflammatory effects can be reached when targeting PPAR*γ* therapeutically. An important example of an anti-inflammatory acting drug, which exerts agonistic effects on PPAR-*γ* and which is widely used in the therapy of especially UC is 5-aminosalicylic acid (5-ASA) [22].

Former studies, which investigated the impact of a polymorphic expression of PPAR*γ* on diseases characterized by proinflammatory processes, focused specifically only on the analysis of the two commonly occurring *NR1C3* SNPs* rs1801282 *and/or *rs3856806.* Several investigations showed that these *NR1C3* gene variants are putatively associated with a moderately higher risk for the development of lifestyle-associated diseases (e.g., metabolic syndrome, coronary artery disease, and type 2 diabetes) or for colorectal cancer [23–29]. However, these findings were only partly supported in subsequent meta-analyses [30–32].

In the study presented here, we aimed at comprehensively investigating the occurring polymorphisms within the *PPAR*γ** gene by sequencing all exonic regions and neighbouring intronic sequences. We analysed the frequency of arising genotypes and haplotypes in a large cohort of IBD patients and non-IBD control subjects and investigated the impact of the observed *NR1C3* variants on IBD susceptibility and disease course.

Interestingly, *NR1C3 *appears to be strongly conserved. Only the two genetic variants *rs1801282 *and *rs3856806, *which have already been described in the literature and which are characterized by a moderately strong linkage behaviour, were found to occur in an allelic frequency of >1%. This observation, together with the fact that all other detected *NR1C3* gene mutations occurred alone in >99% of all cases and never in combination with other *PPAR*γ** gene variants in any individual included in our study, supports the important physiological function of PPAR*γ*, which apparently does not allow a highly polymorphic expression of this protein.

We did not find any significant association of distinct *NR1C3* haplotypes with higher IBD susceptibility or with a modified IBD course. A few other studies have hitherto investigated the impact of a polymorphic PPAR*γ* expression on IBD susceptibility. These studies focused mainly on the investigation of the Pro12Ala polymorphism (*rs1801282*) and its putative influence on UC disease risk. These studies showed heterogeneous results. While [33] observing a significantly higher frequency of homozygous Pro12Ala SNP carriers in UC patients compared to controls in a Danish cohort, Shrestha et al. only observed a putative relationship between a higher UC disease activity and the occurrence of the Pro12Ala variant in a Dutch population, which they could not confirm in a Chinese cohort [34]. A third study focused specifically on the functional impact of the Pro12Ala SNP and showed that this variant appears to be associated with lower *PPAR*γ** mRNA levels in diseased mucosa of UC patients. This finding was combined with a higher prevalence of the Ala-variant in UC patients, when compared to CD patients and healthy controls. The latter observation, however, derives from only a relatively small number of individuals (29 UC and 10 CD patients, 134 controls), which were included in the analysis [35]. Two additional small studies did not find any significant impact of the SNP Pro12Ala on disease susceptibility for CD [36] or UC [37]. In the context of the heterogeneous study outcomes published so far, our study rather supports the hypothesis that a polymorphic expression of the PPAR*γ* gene *NR1C3* does not significantly influence the IBD risk or the course of the IBD forms, CD and UC.

## 5. Conclusions

In conclusion, we have performed a comprehensive study analyzing the role of NR1C3 genetic variants in IBD susceptibility and IBD course in a Swiss cohort of IBD patients. We showed that the polymorphic expression of the *PPAR*γ** gene is not a general modulating risk factor for IBD.

## Supplementary Material

DNA sequences are shown in 5' to 3' direction. F, forward; R, reverse.

## Figures and Tables

**Table 1:** Demographic characteristics of IBD patients and non-IBD controls.

Patient cohort			
	UC	CD	IBD total
*N* (%) of patients	140 (49.3)	144 (50.7)	284 (100)

Male/female *N* (%)	77 (54.6)/64 (45.4)	77 (55)/63 (45)	140 (49.3)/144 (50.7)

Age at enrolment	44.02 ± 14.69	38.59 ± 14.95	41.27 ± 15.04

Disease location *N* (%)	Available for 110 UC patients:	Available for 125 CD patients (Montreal classification)	
	E1: 11 (10%)	L1: 33 (26.4%)	
	E2: 58 (52.7%)	L2: 42 (33.6%)	
	E3: 41 (37.3%)	L3: 46 (36.8%)	
		L4: 4 (3.2%)	

EIM frequency *N* (%)	Available for 139 UC patients:	Available for 142 CD patients:	Available for 281 IBD patients:
	51 (36.7)	72 (50.7)	123 (43.8)

Fistula frequency *N* (%)	Available for 140 UC patients:	Available for 144 CD patients:	Available for 284 IBD patients:
	10 (7.1)	73 (50.7)	83 (29.2)

Mean CDAI at enrolment	NA	Available for 127 CD patients: 110.56 ± 75.81	NA

State of CD (quiescent/acute) *N* (%)	NA	Available for 127 CD pat.:	NA
		95 (74.8)/32 (25.2)	

Mean mtwsi at enrolment	Available for 140 UC patients: 4.69 ± 4.31	NA	NA

State of UC (inactive/active) *N* (%)	118 (84.3)/22 (15.7)	NA	NA

Leucocytes at enrolment	Valid for 134 UC patients: 17.45 ± 79.29	Available for 142 CD patients: 8.9 ± 7.85	Available for 276 IBD patients: 13.05 ± 55.59

CRP at enrolment	Valid for 127 CD patients: 9.58 ± 15.31	Available for 138 CD patients: 12.23 ± 22.64	Available for 265 IBD patients: 10.96 ± 19.48

Control cohort			
			Control total

*N* of controls (%)			194 (100)
Male/female *N* (%)			77 (39.7)/117 (60.3)
Age at enrolment			41.01 ± 16.5

*N*: absolute number; E1: rectal UC; E2: left-sided UC; E3: extensive UC; L1: ileal CD; L2: colonic CD; L3: ileocolonic CD; L4: CD of upper GI; EIMs: extraintestinal manifestations; CDAI: Crohn's activity index; mtwsi: modified Truelove Witts severity index.

**Table 2:** Genetic variants detected in *NR1C3. *

SNP no.	Position in DNA^( a)^	Exon/ intron no.	Exon position	Intron/ exon	Base exchange	Position in RNA^(b)^	AA exchange^(c)^	SNP database
1	12332979	1		Intron	G>T		NA	Not found
2	12333068	1		Exon	insC	68	frame shift	Not found
3	12333070	1		5-prime	C>T	70	5-prime	Not found
4	12333125	1	12333001–12333173	Exon	C>G	125	12 P [Pro]>A [Ala]	rs1801282
5	12333199	1		Intron	T>G		NA	Not found
6	12333213	1		Intron	G>A		NA	Not found
7	12361272	2	12361203–12361430	Exon	C>T	243	51 S [Ser]>F [Phe]	Not found
8	12361422	2		Exon	A>G	393	101 E [Glu]>G [Gly]	Not found
9	12363017	3	12362821–12362990	Intron	A>G		NA	Not found
10	12374014	4		Intron	G>T		NA	Not found
11	12374024	4		Intron	A>T		NA	Not found
12	12374091	4		Intron	C>A		NA	rs4135333
13	12374110	4		Intron	C>T		NA	Not found
14	12374272	4	12374113–12374251	Intron	A>T		NA	rs4135334
15	12374352	4		Intron	C>T		NA	Not found
16	12387616	5	12387381–12387580	Intron	G>A		NA	Not found
17	12398613	6	12398203–12398653	Exon	C>T	1321	410 S [Ser]>S [Ser]	Not found
18	12415473	7		Exon	G>A	1438	449 L [Leu]>L [Leu]	Not found
19	12415557	7	12415397–12415855	Exon	C>T	1522	477 H [His]>H [His]	rs3856806
20	12415581	7		Exon	G>A	1546	485 K [Lys]>K [Lys]	Not found
21	12415647	7		Exon	G>A	1611	506 stop>stop	Not found
22	12415669	7		3′ end	C>T		3′ end	Not found

no.: number; AA: amino acid; ^(a)^DNA reference sequence NT_02257.18; ^ (b)^RNA reference sequence NM_015869.4; ^ (c)^protein reference sequence P37231 (PPARG_HUMAN) SWISSPROT database.

**Table 3:** Allele frequencies in IBD cases and healthy controls.

SNP no.	Position in NT_02257.18*	IBD cases			Controls		
		*N* alleles included	*N *variant alleles	Allele frequency in %	*N* alleles included	*N* variant alleles	Allele frequency in %
1	12332979G>T	566	0	0	364	1	0.27
2	12333068insC	566	1	0.18	364	0	0
3	12333070C>T	566	0	0	364	2	0.55
4	12333125C>G	566	60	10.60	364	50	13.74
5	12333199T>G	566	1	0.18	364	0	0
6	12333213G>A	566	0	0	364	3	0.82
7	12361272C>T	562	1	0.18	358	0	0
8	12361422A>G	562	0	0	358	1	0.28
9	12363017A>G	554	1	0.18	334	0	0
10	12374014G>T	554	1	0.18	348	0	0
11	12374024A>T	564	1	0.18	348	0	0
12	12374091C>A	554	1	0.18	348	0	0
13	12374110C>T	554	1	0.18	348	0	0
14	12374272A>T	554	1	0.18	348	1	0.29
15	12374352C>T	554	0	0	348	1	0.29
16	12387616G>A	562	1	0.18	360	0	0
17	12398613C>T	546	0	0	370	1	0.27
28	12415473G>A	546	0	0	350	1	0.29
19	12415557C>T	546	66	12.09	350	46	13.14
20	12415581G>A	546	0	0	350	1	0.29
21	12415647G>A	546	2	0.37	350	0	0
22	12415669C>T	546	0	0	350	1	0.29

no.: number; *N*: absolute number; *DNA reference sequence signature in NCBI.

**Table 4:** Number of variant carriers in IBD and in non-IBD controls.

SNP	SNP carriers in IBD		SNP carriers in controls		OR (CI)	*P* ^(a)^
	*N* of subjects	*N* (%) of variant carriers	*N* of subjects	*N* (%) of variant carriers		
12332979G>T	283	0 (0)	182	1 (0.5)	NA	0.39
12333068insC	283	1 (0.4)	181	0 (0)	NA	0.61
12333070C>T	283	0 (0)	181	1 (0.6)	NA	0.39
12333125A>G	283	58 (20.5)	182	48 (26.4)	0.72	0.14^(b), (c)^
12333125A>G	227	Hom only: 2 (0.9)	136	Hom only: 2 (1.5)	0.60	0.63^(c)^
12333199T>G	283	1 (0.4)	181	0 (0)	NA	0.61
12333213G>A	283	0 (0)	182	3 (1.6)	NA	0.06
12361272C>T	281	1 (0.4)	179	0 (0)	NA	0.61
12361422A>G	281	0 (0)	179	1 (0.6)	NA	0.39
12363017A>G	277	1 (0.4)	167	0 (0)	NA	0.62
12374014G>T	280	1 (0.4)	174	0 (0)	NA	0.62
12374024A>T	280	1 (0.4)	174	0 (0)	NA	0.62
12374091C>A	280	1 (0.4)	174	0 (0)	NA	0.62
12374110C>T	280	1 (0.4)	174	0 (0)	NA	0.62
12374272A>T	280	1 (0.4)	174	1 (0.6)	0.62	0.62
12374352C>T	280	0 (0)	174	1 (0.4)	NA	0.38
12387616G>A	282	1 (0.4)	179	0 (0)	NA	0.61
12398613C>T	281	0 (0)	185	1 (0.5)	NA	0.40
12415473G>A	273	0 (0)	175	1 (0.6)	NA	0.39
12415557C>T	273	63 (23.1)	175	42 (24)	0.95	0.82^(b ), (c)^
12415557C>T	213	Hom only: 3 (1.4)	137	Hom only: 4 (2.9)	0.48	0.44^(c)^
12415581G>A	273	0 (0)	175	1 (0.6)	NA	0.39
12415647G>A	273	2 (0.7)	175	0 (0)	NA	0.37
12415669C>T	273	0 (0)	175	1 (0.6)	NA	0.39

OR: odds ratio; CI: confidence interval; no.: number; *N*: absolute number; het: heterozygous; hom: homozygous; NA: not applicable; ^(a)^ *P* values calculated with Fisher's exact test; ^ (b)^ *P* value calculated with Chi-Square test; ^(c)^12333125A>G: adjusted for age and sex: *P* = 0.14 (OR 0.72 C.I. (0.46–1.11)) (het + hom); *P* = 0.53 (hom. only); 12415557C>T: adjusted for age and sex: *P* = 0.76 (OR 0.93 C.I. (0.59–1.46)) (het + hom), *P* = 0.35 (OR 0.7 C.I. (0.33–1.49)) (hom only).

**Table 5:** Number of variant carriers (12333125A>G and 12415557C>T) in CD and UC patients compared to controls.

Category				12333125A>G			
	Genotype	*N* ^(a)^	*N* (%) of SNP carriers	OR (C.I.)	*P* ^(c)^	OR (C.I)^ (b)^	*P* ^(b)^
CD	Het plus hom	143	28 (19.6)	0.68 (0.40–1–15)	0.15^(d)^	0.67 (0.40–1.14)	0.14
control	Het plus hom	182	48 (26.4)				
CD	hom	116	1 (0.9)	0.58 (0.05–6.51)	1.0	0.77 (0.23–2.61)	0.68
control	hom	136	2 (1.5)				

UC	Het plus hom	140	30 (21.4)	0.76 (0.45–1.28)	0.31^(d)^	0.78 (0.46–1.32)	0.35
control	Het plus hom	182	48 (26.4)				
UC	hom	111	1 (0.9)	0.61 (0.06–6.81)	1.0	0.70 (0.21–2.38)	0.57
control	hom	136	2 (1.5)				

				12415557C>T			

CD	Het plus hom	139	31 (22.3)	0.91 (0.54–1.54)	0.72^(d)^	0.92 (0.54–1.56)	0.74
control	Het plus hom	175	42 (24.0)				
CD	hom	109	1 (0.9)	0.31 (0.03–2.80)	0.39	0.54 (0.18–1.62)	0.27
control	hom	137	4 (2.9)				

UC	Het plus hom	134	32 (23.9)	0.99 (0.59–1.68)	0.98^(d)^	0.94 (0.55–1.62)	0.83
control	Het plus hom	175	42 (24.0)				
UC	hom	104	2 (1.9)	0.65 (0.12–3.63)	0.70	0.84 (0.35–1.99)	0.69
control	hom	137	4 (2.9)				

OR: odds ratio; CI: confidence interval; *N*: absolute number; het: heterozygous; hom: homozygous; ^(a)^absolute number of all patients of the respective subgroup included into the analysis; ^ (b)^adjusted for age and sex; ^ (c)^ *P* values calculated with Fisher's exact test; ^ (d)^ *P* value calculated with Chi-Square test.

**Table 6:** Impact of variant 12333125A>G on disease activity.

Category				12333125A>G			
	Genotype	*N* ^(a)^	*N* (%) of SNP carriers	OR (C.I.)	*P*	OR (C.I.)^ (b)^	*P* ^(b)^
Fistula	Het plus hom	83	15 (18.1)	1.24 (0.64–2.39)	0.52	1.25 (0.65–2.4)	0.51
No Fistula	Het plus hom	200	43 (21.5)				
Fistula	hom	68	0 (0.0)	NA	1^(c)^	NA	1
No Fistula	hom	159	2 (1.3)				

EIM	Het plus hom	122	23 (18.9)	1.23 (0.68–2.21)	0.5	1.22 (0.67–2.21)	0.51
No EIM	Het plus hom	158	35 (22.2)				
EIM	hom	100	1 (1.0)	0.81 (0.05–13.03)	1^(c)^	0.87 (0.22–3.54)	0.85
No EIM	hom	124	1 (0.8)				

Nonactive UC	Het plus hom	118	26 (22.0)	0.79 (0.25–2.53)	0.79	0.89 (0.27–2.96)	0.85
active UC^(d)^	Het plus hom	22	4 (18.2)				
NonActive UC	hom	93	1 (1.1)	NA	1^(c)^	NA	1
active UC	hom	18	0 (0.0)				

Quiescent CD	Het plus hom	94	17 (18.1)	1.51 (0.58–3.93)	0.4	1. 47 (0.56–3.88)	0.44
Acute CD^(d)^	Het plus hom	32	8 (25.0)				
Quiescent CD	hom	78	1 (1.3)	NA	1^(c)^	NA	1
Acute CD	hom	24	0 (0.0)				

*N*: absolute number; OR: odds ratio; CI: confidence interval; het: heterozygous; hom: homozygous; NA: not applicable; EIMs: extraintestinal manifestations ^(a)^absolute number of subjects included into the respective analysis; ^ (b)^OR and *P* value adjusted for age and sex; ^(c)^ *P* value calculated with Fisher's exact test (otherwise Chi- Square test was used); ^(d)^a threshold of CDAI = 150, and an mtwsi of 10 points was evaluated as the beginning of active disease.

**Table 7:** Impact of variant 12415557C>T on disease activity.

Category	12415557C>T						
	Genotype	*N* ^(a)^	*N* (%) of SNP carriers	OR (C.I.)	*P*	OR (C.I.)^ (b)^	*P* ^(b)^
Fistula	Het plus hom	79	18 (22.8)	1.02 (0.55–1.9)	0.94	1.04 (0.56–1.95)	0.89
No Fistula	Het plus hom	194	45 (23.2)				
Fistula	hom	62	1 (1.6)	0.89 (0.07–9.2)	1^(c)^	0.89 (0.26–3.01)	0.85
No Fistula	hom	151	2 (1.3)				

EIM	Het plus hom	116	26 (22.4)	1.10 (0.62–1.94)	0.76	1.09 (0.61–1.93)	0.78
No EIM	Het plus hom	154	37 (24.0)				
EIM	hom	92	2 (2.2)	0.39 (0.03–4.31)	0.58	0.65 (0.19–2.2)	0.49
No EIM	hom	118	1 (0.8)				

Nonactive UC	Het plus hom	114	28 (24.6)	0.77 (0.24–2.49)	0.78	0.89 (0.27–2.99)	0.86
active UC	Het plus hom	20	4 (20.0)				
Nonactive UC	hom	87	1 (1.1)	5.30 (0.32–90–42)	0.3	3.28 (0.74–14.49)	0.12
active UC	hom	17	1 (5.9)				

Quiescent CD	Het plus hom	91	21 (23.1)	1.16 (0.45–2.97)	0.76	1.22 (0.47–3.17)	0.68
Acute CD	Het plus hom	31	25.8 (31)				
Quiescent CD	hom	71	1 (1.4)	NA	1^(c)^	NA	1
Acute CD	hom	23	0 (0)				

*N*: absolute number; OR: odds ratio; CI: confidence interval; het: heterozygous; hom: homozygous; NA: not applicable; ^(a)^absolute number of subjects included into the respective analysis; ^ (b)^OR and *P* value adjusted for age and sex; ^(c)^ *P* value calculated with Fisher's exact test (otherwise Chi-Square test was used).

**Table 8:** Haplotype and diplotype distribution in IBD cases and non-IBD controls.

	Subjects included	H1 (CC)^(a)^	H2 (CT)	H3 (GC)	H4 (GT)	*P* ^(*c*)^
IBD *N* (%)	256	436.3 (85.2)	41.3 (8.1)	12.7 (2.5)	21.7 (4.2)	0.23
OR (C.I.)		1.27 (0.87–1.86)	0.86 (0.52–1.42)	0.5 (0.23–1.07)	1.1 (0.53–2.28)	
UC *N* (%)	126	212.6 (84.4)	22.6 (9)	6.4 (2.5)	10.4 (4.1)	0.52
OR (C.I.)		1.19 (0.76–1.87)	0.96 (0.54–1.72)	0.51 (0.2–1.3)	1.07 (0.45–2.51)	
CD *N* (%)	130	223.6 (86)	18.6 (7.2)	6.4 (2.5)	11.4 (4.4)	0.31
OR (C.I.)		1.36 (0.86–2.15)	0.75 (0.41–1.39)	0.49 (0.19–1.26)	1.13 (0.49–2.6)	
Controls	148	242.5 (81.9)	27.5 (9.3)	14.5 (4.9)	4.5 (3.9)	

		D1 (CC/CC)^ (b)^	D2 (CC/GT)	D3 (CC/GC)	D4 (CC/CT)	

IBD *N *(%)	256	184.0 (71.9)	37.3 (14.6)	12.0 (4.7)	19.0 (7.4)	
OR (C.I.)		1.3 (0.84–2.02)	0.86 (0.49–1.5)	0.51 (0.23–1.15)	1.24 (0.55–2.81)	
UC *N* (%)	126	89.0 (70.6)	19.6 (15.6)	6.0 (4.8)	9.0 (7.1)	
OR (C.I.)		1.23 (0.73–2.05)	0.93 (0.49–1.78)	0.52 (0.19–1.41)	1.19 (0.46–3.09)	
CD *N* (%)	130	95.0 (73.1)	17.6 (13.5)	6.0 (4.6)	10.0 (7.7)	
OR (C.I.)		1.39 (0.83–2.32)	0.79 (0.41–1.54)	0.5 (0.19–1.36)	1.29 (0.51–3.27)	
Controls	148	98.0 (66.2)	24.5 (16.6)	13.0 (8.8)	9.0 (6.1)	

		D5 (CT/GT)	D6 (GC/GT)	D7 (GC/CT)	D8 (GT/GT)	*P* ^(*c*)^

IBD *N*(%)		2.0 (0.8)	0.0 (0)	0.7 (0.3)	1.0 (0.4)	0.409
OR (C.I.)		0.58 (0.08–4.12)	—	—	—	
UC *N* (%)		1.0 (0.8)	0.0 (0)	0.0 (0)	1.0 (0.8)	0.716
OR (C.I.)		—	—		—	
CD *N* (%)		0.0 (0)	0.0 (0)	0.0 (0)	0.0 (0)	0.588
OR (C.I.)		—	—		—	
Controls		2.0 (1.4)	1.0 (0.7)	0.5 (0.3)^ (d)^	0.0 (0)	

OR: odds ratio; CI: confidence interval; *N*: absolute number; H1–H4: haplotype 1—haplotype 4; D1–D8: diplotype 1—diplotype 8; ^(a)^the first base denotes the outcome at position *rs1801282*, the second base denotes the outcome at position *rs1801282*; ^(b)^each base pair (before and after the slash) denotes one haplotype; ^(c)^ *P* values calculated with FAMHAP; ^(d)^frequency predicted to be 0.0 when comparing UC patients with controls.

**Table 9:** Haplotype and diplotype distribution in IBD cases with and without EIMs.

	Subjects included	H1 (CC)	H2 (CT)	H3 (GC)	H4 (GT)	*P* ^(a)^
IBD *N *(%)	110	189.8 (86.2)	10.2 (4.6)	3.2 (1.5)	16.8 (7.6)	0.57
OR (CI)		1.18 (0.72–1.95)	1.18 (0.50–2.80)	0.44 (0.12–1.58)	0.87 (0.46–1.67)	
Controls^(b)^	143	240.7 (84.1)	11.4 (4.0)	9.3 (3.3)	24.7 (8.6)	
UC *N* (%)	45	76.9 (85.5)	5.1 (5.6)	1.1 (1.2)	6.9 (7.7)	0.55
OR (CI)		1.15 (0.56–2.37)	1.79 (0.51–6.25)	0.36 (0.04–2.93)	0.76 (0.3–1.93)	
Controls^(b)^	80	133.8 (83.6)	5.2 (3.2)	5.2 (3.2)	15.8 (9.9)	
CD *N* (%)	65	112.8 (86.8)	5.2 (4.0)	2.2 (1.7)	9.8 (7.6)	0.82
OR (CI)		1.18 (0.58–2.38)	0.8 (0.24–2.66)	0.49 (0.09–2.61)	1.09 (0.42–2.79)	
Controls^(b)^	63	106.8 (84.8)	6.2 (4.9)	4.2 (3.3)	8.8 (7.0)	

		D1 (CC/CC)	D2 (CC/GT)	D3 (CC/GC)	D4 (CC/CT)	*P* ^(a)^

IBD *N* (%)	110	82.0 (74.5)	14.8 (13.4)	3.0 (2.7)	8.0 (7.3)
OR (CI)		1.30 (0.75–2.27)	0.82 (0.41–1.67)	0.42 (0.11–1.58)	0.94 (0.37–2.43)
Controls^(b)^	143	99.0 (69.2)	22.7 (15.8)	9.0 (6.3)	11.0 (7.7)	
UC *N* (%)	45	33.0 (73.3)	(13.2)	1.0 (2.2)	4.0 (8.9)
OR (CI)		1.25 (0.55–2.82)	0.73 (0.26–2.06)	0.34 (0.04–3.01)	1.46 (0.37–5.75)
Controls^(b)^	80	55.0 (68.8)	13.8 (17.3)	5.0 (6.3)	5.0 (6.3)	
CD *N* (%)	65	49.0 (75.4)	8.8 (13.6)	2.0 (3.1)	4.0 (6.2)
OR (CI)		1.32 (0.61–2.88)	0.96 (0.35–2.63)	0.47 (0.08–2.65)	0.62 (0.17–2.32)
Controls^(b)^	63	44.0 (69.8)	8.8 (14.0)	4.0 (6.3)	6.0 (9.5)	

		D5 (CT/GT)	D6 (GC/GT)	D7 (GC/CT)	D8 (GT/GT)	

IBD *N *(%)		2.0 (1.8)	NP	NP	0.0 (0)	0.36
OR (CI)		—			—	
Controls		0.0 (0)	NP	NP	1.0 (0.7)	
UC *N* (%)		1.0 (2.2)	NP	NP	0.0 (0)	0.6
OR (CI)		—			—	
Controls		0.0 (0)	NP	NP	1.0 (1.3)	
CD *N *(%)		1.0 (1.5)	NP	NP	NP	0.73
OR (CI)		—				
Controls		0.0 (0)	NP	NP	NP	

OR: odds ratio; CI: confidence interval; *N*: absolute number; H1–H4: haplotype 1—haplotype 4; D1–D8: diplotype 1—diplotype 8; EIMs: extraintestinal manifestations; NP: predicted not to appear in the case or control group; ^(a)^ *P* values calculated with FAMHAP; ^(b)^the term “controls” denotes here the respective patient subgroup (IBD/UC/CD) without EIMs.

**Table 10:** Haplotype and diplotype distribution in IBD cases with and without fistulas.

	Subjects included	H1 (CC)	H2 (CT)	H3 (GC)	H4 (GT)	*P* ^(a)^
IBD *N* (%)	77	132.9 (86.3)	7.1 (4.6)	3.1 (2.0)	10.9 (7.0)	0.89
OR (C.I.)		1.13 (0.65–1.94)	1.16 (0.46–2.9)	0.77 (0.21–2.81)	0.81 (0.40–1.67)	
Controls^ (b)^	179	303.6 (84.8)	14.4 (4.0)	9.4 (2.6)	30.6 (8.5)	
UC *N* (%)	9	17.0 (94.4)	0.0 (0.001)	0.0 (0.001)	1.0 (5.5)	0.65
OR (C.I.)			0.02 (0.0–439897.28)	0.03 (0.0–739520.9)	0.57 (0.07–4.52)	
Controls^ (b)^	117	195.8 (83.7)	10.2 (4.4)	6.2 (2.7)	21.8 (9.3)	
CD *N *(%)	68	115.8 (85.2)	7.2 (5.3)	3.2 (2.3)	9.8 (7.2)	0.90
OR (C.I.)		0.86 (0.43–1.74)	1.6 (0.47–5.51)	0.91 (0.19–4.42)	1.02 (0.40–2.61)	
Controls^ (b)^	72	107.8 (87.0)	4.2 (3.3)	3.2 (2.5)	8.8 (7.1)	

		D1 (CC/CC)	D2 (CC/GT)	D3 (GC/CT)	D4 (CC/CT)	*P* ^(a)^

IBD *N *(%)	77	57.0 (74.0)	9.9 (12.8)	NP	6.0 (7.8)
OR (C.I.)		1.17 (0.64–2.13)	0.81 (0.37–1.76)		1.08 (0.39–2.95)
Controls^ (b)^	179	127.0 (70.9)	27.6 (15.4)	NP	13.0 (7.3)	
UC *N* (%)	9	8.0 (88.9)	1.0 (11.0)	NP	0.0 (0)
OR (C.I.)		3.56 (0.43–29.49)	0.65 (0.08–5.53)		—
Controls^ (b)^	117	81.0 (69.2)	18.8 (16.0)	NP	9.0 (7.7)	
CD *N* (%)	68	49.0 (72.1)	8.8 (13.0)	NP	6.0 (8.8)
OR (C.I.)		0.90 (0.41–1.95)	0.90 (0.33–2.45)		1.40 (0.38–5.23)
Controls^ (b)^	72	46.0 (74.2)	8.8 (14.3)	NP	4.0 (6.5)	

		D5 (CC/GC)	D6 (CT/GT)	D7 (GT/GT)	D8 (GC/GT)	

IBD *N* (%)		3.0 (3.9)	1.0 (1.3)	0.0 (0)	NP	0.98
OR (C.I.)		0.77 (0.20–2.91)	—	—		
Controls		9.0 (5.0)	1.0 (0.6)	1.0 (0.6)	NP	
UC *N *(%)		0.0 (0)	0.0 (0)	0.0 (0)	NP	0.67
OR (C.I.)		—	—	—		
Controls		6.0 (5.1)	1.0 (0.9)	1.0 (0.9)	NP	
CD *N* (%)		3.0 (4.4)	1.0 (1.5)	NP	NP	0.98
OR (C.I.)		0.91 (0.18–4.67)	—			
Controls		3.0 (4.8)	0.0 (0)	NP	NP	

OR: odds ratio; CI: confidence interval; *N*: absolute number; H1–H4: haplotype 1—haplotype 4; D1–D8: diplotype 1—diplotype 8; NP: predicted to not appear in the respective case and control group; ^(a)^ *P* values calculated with FAMHAP; ^(b)^the term “controls” denotes here the respective patient subgroup (IBD/UC/CD) without the appearance of fistulas.

**Table 11:** Haplotype and diplotype distribution in IBD cases with and without disease activity.

	Subjects included	H1 (CC)	H2 (CT)	H3 (GC)	H4 (GT)	*P* ^(a)^
IBD *N *(%)	48	82.9 (84.6)	3.1 (3.2)	2.1 (2.2)	9.9 (10.1)	0.83
OR (C.I.)		0.99 (0.53–1.83)	0.65 (0.19–2.21)	0.88 (0.2–3.96)	1.29 (0.6–2.74)	
Controls^ (b)^	191	323.6 (84.7)	18.4 (4.8)	9.4 (2.5)	30.6 (8.0)	
UC *N* (%)	18	31.0 (86.0)	1.0 (2.9)	0.0 (0.1)	4.0 (11.0)	0.74
OR (C.I.)		1.16 (0.42–3.18)	0.67 (0.08–5.23)	0.04 (0.0–749.8)	1.3 (0.41–4.09)	
Controls^ (b)^	108	181.8 (84.2)	9.2 (4.3)	6.2 (2.9)	18.8 (8.7)	
CD *N* (%)	31	51.9 (83.7)	2.1 (3.4)	2.1 (3.4)	5.9 (9.5)	0.72
OR (C.I.)		0.88 (0.39–1.95)	0.6 (0.13–2.76)	1.79 (0.31–10.5)	1.37 (0.49–3.86)	
Controls^ (b)^	83	141.8 (85.4)	9.2 (5.5)	3.2 (1.9)	11.8 (7.1)	

		D1 (CC/CC)	D2 (CC/GT)	D3 (GC/CT)	D4 (CC/CT)	*P* ^(a)^

IBD *N *(%)	48	35.0 (71.4)	8.9 (18.1)	NP	2.0 (4.1)
OR (C.I.)		1.04 (0.52–2.08)	1.31 (0.57–3.01)		0.44 (0.10–1.95)
Controls^ (b)^	191	135.0 (70.7)	27.6 (14.4)	NP	17.0 (8.9)	
UC *N* (%)	18	14.0 (77.8)	3.0 (16.4)	NP	0.0 (0)	
OR (C.I.)		1.54 (0.47–5.03)	1.07 (0.28–4.13)		—	
Controls^ (b)^	108	75.0 (69.4)	16.8 (15.5)	NP	9.0 (8.3)	
CD *N* (%)	31	21.0 (67.7)	5.9 (19.0)	NP	2.0 (6.5)
OR (C.I.)		0.81 (0.33–1.97)	1.57 (0.52–4.72)		0.65 (0.13–3.23)
Controls^ (b)^	83	60.0 (72.3)	10.8 (13.0)	NP	8.0 (9.6)	

		D5 (CC/GC)	D6 (CT/GT)	D7 (GT/GT)	D8 (GC/GT)	

IBD *N *(%)		2.0 (4.1)	1.0 (2.0)	0.0 (0)	NP	0.70
OR (C.I.)		0.86 (0.18–4.12)	—	—		
Controls		9.0 (4.7)	1.0 (0.5)	1.0 (0.5)	NP	
UC *N* (%)		0.0 (0)	1.0 (5.6)	0.0 (0)	NP	0.16
OR (C.I.)		—	—	—		
Controls		6.0 (5.6)	0.0 (0)	1.0 (0.9)	NP	
CD *N* (%)		2.0 (6.5)	0.0 (0)	NP	NP	0.80
OR (C.I.)		1.84 (0.29–11.57)	—			
Controls		3.0 (3.6)	1.0 (1.2)	NP	NP	

OR: odds ratio; CI: confidence interval; *N*: absolute number; H1–H4: haplotype 1—haplotype 4; D1–D8: diplotype 1—diplotype 8; NP: predicted to not appear in the respective case or control group; ^(a)^ *P* values calculated with FAMHAP; ^(b)^the term “controls” denotes here the respective patient subgroup (IBD/UC/CD) without the respective ongoing disease activity pattern investigated.

## Abbreviations

IBD: Inflammatory bowel disease
UC: Ulcerative colitis
CD: Crohn's disease
PPAR*γ*: Peroxisome proliferator-activated receptor gamma
SNP: Single nucleotide polymorphism
NR1C3: Nuclear receptor subfamily 1, group C, member 3
EIMs: Extraintestinal manifestations.
MTWSI: Modified truelove witts severity index
CDAI: Crohn's disease activity index.s
