# Supplementary material for: The Impact of PPARγ Genetic Variants on IBD Susceptibility and IBD Disease Course
Source: PPAR Res. 2012 Feb 14;2012:349469. doi: 10.1155/2012/349469 (PMC3289871; doi:10.1155/2012/349469)
Supplement: Supplementary file 1 — DNA sequences are shown in 5' to 3' direction. F, forward; R, reverse. [file 349469.f1.pdf]

Supplemental Table 1: Oligonucleotides used as PCR primers to amplify the *NR1C3* exons.

Primer name	Primer sequence	Nested PCR	Primer name	Primer sequence
PPARg 1_F	ttcaagcccagtcctttctg	no		
PPARg 1_R	aaatgaacgcgatagcaacg	no		
PPARg 3_F	tcataaaacagcctagacagcac	no		
PPARg 3_R	cccttcctggcatttcatag	no		
PPARg 4_F	tgcctgtgattacaaacctttc	yes	PPARg 4_Fa	tgggggtcatagaaccacag
PPARg 4_R	ggctgcagtgtgctaggatag	yes	PPARg 4_Ra	gatcacttgagcccagaagc
PPARg 5_F	agggacctggagatcctctg	no		
PPARg 5_R	agatgcccaaccacagagag	no		
PPARg 6_F	tgatggtctgtgctacttttgtg	no		
PPARg 6_R	ttgtgctatctgaaaacacttcc	no		
PPARg 7_F	ctcattattaagcatcttcagc	no		
PPARg 7_R	tcacaccgcaaacctatgac	no		
PPARg 8_F	tcttacccattatctgcttacc	no		
PPARg 8_R	gtgtctttataaacaatatgc	no		

DNA sequences are shown in 5’ to 3’ direction.
F, forward; R, reverse.
